# The Processes of Nutrition and Metabolism Affecting the Biosynthesis of Milk Components and Vitality of Cows with High- and Low-Fat Milk

**DOI:** 10.3390/ani12050604

**Published:** 2022-02-28

**Authors:** Evgeniy Kharitonov

**Affiliations:** All-Russia Research Institute of Animal Physiology, Biochemistry, and Nutrition—Branch of the Federal Science Center for Animal Husbandry Named after Academy Member L. K. Ernst, 249010 Borovsk, Russia; evgenijkharito@yandex.ru

**Keywords:** cows, milk fat, rumen fluid, milk fatty acids, energy balance

## Abstract

**Simple Summary:**

Rumen fermentation, the level of hormones and major metabolites in the blood, and tissue heat production, as well as milk composition, were studied in high-producing cows with different milk fat content. It was found that low milk fat is associated with neither a lack of acetate formation in the rumen, nor a change in the hormonal profile. The result is an increase in the availability of cows for metabolizable energy, the likelihood of ketosis, and the extension of their productive use.

**Abstract:**

In order to clarify the mechanism of the depression of milk fat formation and preserve the health of animals, the aim of the research was to study the characteristics of rumen digestion, energy metabolism, and milk composition in high-producing dairy cows with high and low levels of milk fat that are fed the same diet. Two groups of cows with normal milk fat content (3.94 ± 0.12; *n =* 10) and low milk fat content (2.95 ± 0.14, *n* = 10) contained in the same diet were identified. Gas exchange (O_2_ uptake and CO_2_ output) was studied in cows and blood samples, rumen contents (pH, NH_3_-N), and VFA and milk (fat, protein, and fatty acid composition) were collected and analyzed. It was determined that cows with low fat milk are more efficient at using the metabolized energy of their diets due to the tendency to have a decrease in the proportion of heat production (by 6.2 MJ; *p* = 0.055) and an earlier start of a positive energy balance. At the same time, the fat content in milk did not depend on the level of hormones in the blood or on the formation of acetate in the rumen. An analysis of the duration of the productive use of cows on this farm (*n* = 650) showed that the number of lactations was inversely correlated with the level of fat in milk (r = −0.68; *p* < 0.05, *n* = 1300). These results indicate the advantages of cows that can reduce the fat content of their milk in the first months of lactation.

## 1. Introduction

Maintaining the normal physiological state of animals is the key to their long-term productive use and the guarantee of obtaining high-quality milk. Only a healthy cow is able to provide high productivity. Therefore, the health of the cow and her productivity are inextricably linked. In recent years, the number of diseases of alimentary etiology has increased significantly. The main breakdown occurs in the first months of lactation, when the feed consumption potential lags behind the growth rate of milk production [1].

As a result, there is an increased mobilization of fat depots to reduce the negative energy balance in the body of cows. Therefore, in the first phase of lactation, the use of concentrated feeds in large quantities is practiced. The main negative aspect when feeding high-concentration diets is a decrease in rumen pH, which causes a change in rumen metabolism accompanied by the acidification of the contents of the rumen, a violation of microbial activity, a decrease in acetate formation, a drop in milk fat, and a number of other negative consequences associated with subacute rumen acidosis, or SARA (acidosis, laminitis, and infertility). The economic losses associated with SARA consist of a shortage of dairy products, a decrease in quality, the costs of veterinary measures, losses associated with the death or forced slaughter of animals, a decrease in reproduction functions, complications at the hotel, etc. For the health of the herd, the most dangerous impact of SARA is subclinical acidosis, for which there are no obvious signs, and the consequences manifest after a significant delay. A diet in which the rumen has a pH of medium to lower than 6.0 can cause the depression of fat formation, and the addition of buffers to correct milk fat depression is an effective means to increase pH and milk fat in such situations [2].

An inadequate amount of fiber results in a low pH in the rumen. Low rumen pH affects the ratio of trans-10 fatty acids produced by biohydrogenation and potentially inhibits the complete saturation of trans-18: 1 fatty acids to stearins. We found that an increase in trans-10 fatty acids in milk fat and a decrease in milk fat appear only when diets with a low level of roughage (low rumen pH) supplemented with vegetable oil (a source of polyunsaturated fatty acids (PUFA)) are fed [3]. A direct effect of rumen pH was shown when the addition of buffers to a high-concentration diet reduced duodenal trans-fatty acids (TFA) flow and increased fat [4]. The addition of high levels of PUFA to a normal roughage diet has been ineffective in causing a depression of milk fat [5]. Diets formulated with adequate fiber to maintain a normal pH and those that also limit PUFAs in the feed as potential TFAs will contribute to a normal percentage of fat in milk [5].

A reduction of energy deficiency in the first stage of lactation is achieved by the use of special fat additives that do not affect microbial fermentation in the rumen [6,7]. Reducing the energy deficit in cows at the beginning of lactation can be achieved by reducing the release of energy from milk by reducing the fat content in it. For these purposes, additives containing mixtures of positional and geometric isomers of octadecadienoic acid with conjugated bonds (conjugate linoleic acid (CLA)) have been studied and used [8,9,10,11]. It has been demonstrated that feeding such additives protected cows from the effects of rumen microorganisms and reduced the fat in milk, depending on the dose, from 36 to 62%, and, at the same time, did not affect milk yield [12]. Diets high in starch and low in fat, which can cause subacute rumen acidosis, may temporarily reduce milk fat synthesis [13].

In order to clarify the mechanism of the depression of milk fat formation and preserve the health of animals, the aim of the research was to study the characteristics of rumen digestion, energy metabolism, and milk composition in high-productivity cows with high and low levels of milk fat that are fed the same diet.

## 2. Materials and Methods

### 2.1. Experiment Design and Animal Management

The studies were carried out on cows of the Holstein breed during their second lactation at the end of the first phase (80 days) using milk from the previous lactation (8–9 thousand kg of milk). Based on the data from the controlled milking, milk composition, lactation day, fatness, and lactation number, two groups of cows (10 heads each) were found in the same feeding group (a group of highly productive cows), but with different fat contents of milk (according to the results of the last controlled milking (Table 1)). At the same time, by origin (assigned bull-producer), the cows in the groups did not differ.

The feeding ration for all groups of cows was the same and was set in the form of a feed mixture consistent with the level of milk production, live weight, and day of lactation, and was within the limits of the allowed content of individual nutrients (Table 2). The cows’ diets were represented by silage corn (20 kg), haylage of perennial grasses (8 kg), hay grass (0.5 kg), mixed fodder (16 kg), soybean meal (1 kg), fresh beer pellet (6 kg), and molasses (1 kg).

Cows were also fed compound feed consisting of wheat (20%), barley (33%), oats (5%), maize (13%), sunflower meal (25%), salt (1%), mineral additives (1%), and premix (1%).

Salt plus trace mineralized salt and vitamins were available ad lib and contained 38.5% NaCl and 61.5% of a trace mineral and vitamin mix. Trace mineral and vitamin mix contained 0.11% Mn, 0.14% Zn, 0.05% Fe, 0.025% Cu, 0.0027% I, 0.0024% Co, and 0.0007% Se. It also contained 650,000 IU of vitamin A, 250,000 IU of vitamin D3, and 2500 IU of vitamin E per kg of mix.

### 2.2. Sample Collection and Measurement

#### 2.2.1. Rumen Fluid Collection and Measurement

Samples of rumen liquid were obtained 3 h after morning feeding using an oral gastric tube. The initial 100 mL of rumen fluid was discarded to avoid saliva contamination, and, finally, 100 mL of rumen fluid was filtered by four layers of cheesecloth and then collected. The rumen fluid pH was immediately measured using a pH electrode (OHAUS Starter ST2100-B) after collection. Immediately after rumen fluid collection, a 10 mL aliquot of ruminal fluid supernatant was preserved by adding 1 mL of 25% metaphosphoric acid for volatile fatty acid (VFA) determination, and another 50 mL aliquot of untreated ruminal fluid was kept for other analysis.

The NH_3_-N concentration was measured using a microdiffusion method [14] in Conway dishes. Laboratory studies of the enzymatic activity of the microflora of the animal rumen were carried out using glass capillaries (in vitro). Cellulolytic activity was evaluated in accordance with the Henderson method [15] by the difference in the weight of the threads before and after incubation. The amylolytic ability of microorganisms in relation to “pure” nutrients (a 20% solution of potato starch) was studied. The amount of enzymatic activity was judged by the decrease in starch concentration in the solution after a 24 h incubation period. Quantitative indicators of rumen microbiocenosis were determined in the Goryaev chamber for 15–20 min from the time of collection, and the number of ciliates and the total number of bacteria in 1 mL of the contents were counted according to standard procedures [16].

Samples of ruminal fluid were analyzed for VFA using a gas chromatograph (Cvet–800, Rus) equipped with a Hromaton N-AWDMCS (Chech) fused-silica capillary column (1.5 m × 3 mm i.d.). The detector temperature was 250 °C, the hydrogen carrier gas flow to the detector was 30.0 mL/min, airflow was 300 mL/min, and the flow of nitrogen makeup gas was 30.0 mL/min. A volatile free-acid mixture standard (MTY-1075 Matreya LLG, USA) was used for VFA determination.

#### 2.2.2. Indicators of Energy Metabolism in Cows

Heat production was measured using the method of indirect calorimetry in respiratory trials using face masks [17]. The study of pulmonary gas exchange by the mask method allowed us to obtain a number of indicators characterizing pulmonary respiration, gas exchange, and tissue energy costs, such as: the volume of pulmonary ventilation per unit of time, in liters; respiratory rate per minute; exhalation capacity, in liters; the concentration of absorbed oxygen from inhaled air, as a percentage; the concentration of carbon dioxide in exhaled air, as a percentage; and the total volume of absorbed oxygen and released carbon dioxide during the experiment. Based on these data, the respiratory coefficient was calculated, the caloric value of one liter of oxygen consumed was determined, and the energy costs in the animals were calculated. Immediately afterward, the exhaled gases collection analysis of 0_2_ and C0_2_ was carried out on a gas analyzer-chromatograph (AHT-TI, Rus). The gross energy of the diets and milk samples was determined using an adiabatic bomb calorimeter (ABK-1, Rus).

#### 2.2.3. Plasma Samples

Blood samples were obtained 3 h after morning feeding by puncturing the tail artery, and then transferring the samples into vacutainers with sodium citrate. Plasma samples were obtained from the blood samples after centrifugation at 1500× *g* for 15 min at 4 °C. The plasma was then preserved at −20 °C until analysis. In the blood plasma, the glucose (GLU) content was determined with the help of an enzymatic colorimetric glucose oxidase method (using the panel of reagents from the firm Soared Diagnostic, SPb RUS), the concentration of non-esterified fatty acids (NEFA) was determined by means of enzymatic colorimetric method kit (Rendox, Crumlin, UK), β-hydroxybutyrate was assessed (with a set of reagents from Rendox), VFA was analyzed using a gas chromatograph (Cvet–800, Rus), urea was analyzed (with a set the reagents “Urea 450” with diacetylmonooxime from the firm Lahema), α-amine nitrogen [18] was assessed, and triacylglycerols were anylyzed by an enzymatic colorimetric method (with the panel of reagents from the firm Soared Diagnostic, SPb RUS). In the blood plasma samples, the concentration of insulin, thyroxine, triiodothyronine, and cortisol were determined by an enzyme immunoassay using commercial kits (DRG, Marburg, Germany).

In the blood plasma, the activity of pyruvate carboxylase was determined by the method described by Scrutton [19] and by lactate dehyrogenase LDH, wherein a set of Lahem’s company is used when NAD is introduced into the reaction medium to determine the rate of conversion of lactate to pyruvate. The amount of acetoaldehyde was determined at 340 nm on a Specol-11 spectral colorimeter (Carl Zeiss, Oberkochen, Germany) after preliminary preparation. Using a chromatograph (Millichrom, RUS), the amounts of α-tocopherol, retinol, and thiamine were defined.

#### 2.2.4. Milk Composition Analysis

Samples of milk from three consecutive days were combined according to yield for each day and for each cow on the 50th, 80th, 100th, 140th, 170th, and 200th days of lactation. The milk was sampled in a 50 mL tube and analyzed immediately. The samples were analyzed using a milk analyzer (Lactostar, GmbH, Munich, Germany) for protein, fat, lactose, and urea nitrogen content.

#### 2.2.5. Fatty Acid Analysis

Lipids were extracted from the milk according to Folch [20] by using methanol–chloroform (2:1), and then transmethylating. For analysis of FAs, a gas chromatograph (Cvet – 800, Rus) equipped with a Hromaton AWYMDS fused-silica capillary column (3.5 m × 3 mm i.d.) was used. The inlet and flame-ionization detector temperatures were 200 °C, and a 1-μL injection volume was used. The hydrogen carrier gas flow to the detector was 30 mL/min, airflow was 300 mL/min, and the flow of nitrogen makeup gas was 30 mL/min. Fatty acid peaks were identified by using a fatty acid methyl ester standard (SUCPM-47885, Supelko; Bellefonte, PA, USA).

### 2.3. Statistical Analysis

The obtained results were statistically analyzed using the general linear models (GLM) procedure, adapted from the IBM SPSS Statistics 11.5 user’s guide, with one-way ANOVA according to the following model: Yij = µ + xi + eij, where Yij is the dependent variable, µ refers to the mean, xi is the effect of treatment, and eij is the experimental error. Data are expressed as LS means and SE, unless reported otherwise. Differences were assumed to be significant if *p* ≤ 0.05 and *p* > 0.05, but *p* < 0.10 was considered a trend. The correlation analysis between the characteristics of the ruminal fluid and the amount of lactation between the fat of milk was performed using the Spearman procedure via the two-tailed test. *p* ≤ 0.05 were considered statistically significant and 0.05 < *p* < 0.1 was considered a trend difference.

## 3. Results

### 3.1. Ruminal Fermentation Parameters

The study of enzymatic and microbiological processes in the rumen of cows with different milk fat content showed that the indicators of rumen digestion corresponded to the characteristics of the cows’ diets. There were no signs of subacute acidosis (Table 3). The study of enzymatic and microbiological processes in the rumen of cows with different milk fat content showed that the indicators of digestion in the rumen corresponded to the characteristics of the cows’ diets. At the same time, cows with low milk fat content showed a tendency to have decreased pH (*p* = 0.09), fibrolytic activity (*p* = 0.07), a significant decrease in buffering capacity (*p* < 0.05), and an increase in the level of propionate (*p* < 0.05) compared to cows with normal milk fat content. 

The study of the correlation dependencies of rumen metabolism and milk fat content showed a direct dependence on pH (r = 0.62; *p* < 0.05), acetate level (r = 0.64; *p* < 0.05), and the ratio of acetate to propionate (r = 0.82; *p* < 0.05). An inverse relationship was found for propionate (r = −0.73; *p* < 0.05).

### 3.2. Biochemical Parameters of the Blood of the Cows

The analysis of the biochemical parameters of the cows’ blood showed that the level of blood metabolites corresponded to the stage of lactation, the cows’ productivity, and the characteristics of the cows’ diets (Table 4). Analysis of the blood biochemical parameters showed that the level of blood metabolites corresponded to the stage of lactation, the cows’ productivity, and the cows’ dietary characteristics (Table 4). In the blood of cows with low milk fat content, there was a significant decrease in the concentration of non-esterified fatty acids (*p* < 0.05), with a higher level of VFA (*p* < 0.05). This indicates a higher rate of mobilization of the body’s fat depots to ensure the synthesis of milk components.

### 3.3. Indicators of Energy Metabolism in Cows

Analysis of the data on energy exchange showed that cows with low milk fat tended to release less energy with milk (*p* = 0.1), and instead spend it on heat generation (by 6.2 MJ; *p* = 0.055) (Table 5). As a result, cows with low milk fat had a positive energy balance earlier, as evidenced by a significant (26.1 MJ *p* = 0.035) level of energy retention. During the observation of the fatness of cows from the 50th to the 80th days of lactation, it was determined that cows with normal milk fat content lost 1.5 points of fatness, while cows with low milk fat content lost only 1 point (*p* = 0.43), with unreliable differences.

### 3.4. Milk Yield and Compositions

During sampling (days 80–82 of lactation), productivity indicators and milk composition significantly (*p* < 0.05) differed between the groups, though only for milk fat (Table 6). A significant difference was noted between the indicators only for the fat content in milk. Analysis of the milk of the various groups showed that all milk had no deviations in sanitary and hygienic standards, or in technological properties.

#### Fatty Acid Composition of Milk Lipids

The determination of the fatty acid composition of milk lipids showed that only an increased content of fatty acids with an odd number of atoms (C_15_, C_17_) was revealed in the milk of cows with a low milk fat content (*p* < 0.05) (Table 7).

## 4. Discussion

It is generally believed that the formation and concentration of fat in the milk of dairy cows is influenced by diet [21,22,23,24,25]. In our experiment, the cows of both groups received the same diet; however, the fat content of milk was very different. The study of the subsequent milk productivity showed that by the 140th day of lactation, the fat content of the milk of the cows of both groups did not change (Table 8).

Lower milk fat content means that less energy is required for milk synthesis. Romo et al. [26] noted that dietary *trans*-C18:1, which reduces milk fat percentage, significantly decreased milk energy output by 3.4 Mcal/d and numerically increased energy retained in tissue by 3.4 Mcal/d when compared with *cis*-C18:1. This reciprocal change between milk fat synthesis by the mammary gland as compared to fat deposition in adipose tissue has been observed for various dietary situations which cause milk fat depression [21,27,28].

Reducing the fat content of milk in the first phase of lactation by the temporary artificial blocking of fat synthesis is used in practical cattle breeding to prevent excessive loss of body weight and preserve reproductive function. For this purpose, feed additives based on conjugated fatty acids are used. In such earlier experiments carried out [29], it was possible to reduce the release of energy with milk by 13.3% and to reduce the negative energy balance by 6.6 MJ. In the current experiment, the energy yield with milk decreased by 13.2%, and the energy retention increased by 26.1 MJ (*p* < 0.05) (Table 5).

Cows with low milk fat had an earlier onset of positive energy balance and a shorter service period (109.5 ± 19.8 vs. 139 ± 18.4 days; *p* = 0.194). Thus, a decrease in the volume of synthesis of fatty acids in the mammary glands, as the most energy-intensive process, allows cows to reduce metabolic stress and maintain health.

Analysis of the duration of productive use of the cows on this farm showed that the number of lactations was inversely correlated with the level of fat in milk (r = −0.68; *p* < 0.05, *n* = 1300). Special experiments aimed at assessing the long-term effects of feeding diets that reduce fat content have not been conducted, although it is noted that there are enough examples of apparently healthy herds that produce milk with a fat content in the range of 2.5 to 3.0%, which indicates that the level of milk fat is not necessarily incompatible with “healthy” cows [22].

Milk fat depression caused by high concentrate, low forage diets usually occurs within a few days following dietary changes and is characterized by a substantial reduction in both yield and percentage of milk fat. Usually, a change in milk fat content (decrease) is associated with an increased consumption of concentrates and, thus, a low pH value of the rumen contents, a low proportion of acetate, and a decrease in the biohydrogenation capacity of the rumen microflora. High concentrate diets result in increased rumen production of propionate and increased hepatic rates of gluconeogenesis, which in turn increase the pancreatic release of insulin. In our research, we have studied the possible causes of milk fat reduction. Most of the proposed mechanisms that influenced milk fat content were associated with of a limited supply of lipid precursors in VFA content and a corresponding change in VFA metabolism [30]. Therefore, it was believed that low fat in milk is associated with a lack of acetate formation in the rumen as a precursor for the synthesis of fatty acids in the mammary gland because acetate and β -hydroxybutyric acid (BHBA) account for almost all of the carbon in the de novo synthesis of fatty acids by the mammary gland under normal situations, as well as during milk fat depression. However, the content of acetate in the contents of the rumen did not decrease (6.03 mmol/dL and 6.65 mmol/dL in the two groups, respectively) (Table 3). The blood acetate level in cows with low milk fat did not differ from those in cows with normal milk fat (Table 4).

Another precursor for the formation of milk fat is BHBA. In our experiments on the rumen content, the concentration of butyric acid in cows with low milk fat was only slightly lower than in cows with normal milk fat (1.42 ± 0.02 versus 1.32 ± 0.02; *p* > 0.05) (Table 3). At the same time, the content of BHBA in the blood was also slightly lower (*p* > 0.05) (Table 4). The use of radioisotopes has shown that acetate content does not become deficient during the depression of fat formation [31]. It was also concluded that a BHBA deficiency for milk fatty acid synthesis is not a causative factor in the decrease in milk fat production [32]. Results of the present study also indicate that the reduction in milk fat content was not due to a decrease in the production of acetate and BHBA.

Another possible mechanism for reducing fat in milk is related to an increase in insulin in the blood and a deficiency of vitamin B [27]. It was suggested that the decrease in milk fat previously observed with hyperinsulinemic-euglycemic clips, or with glucose [33] or propionate infusions was the most likely consequence of the ability of insulin to inhibit lipolysis, thereby limiting the availability of preformed fatty acids mobilized from body reserves in the mammary glands [34]. The involvement of thyroid hormones in the regulation of milk fat production has been reported [35]. However, neither vitamin [36] nor insulin [37] injections affected milk fat. We found neither cortisol nor thyroid hormones in the content of insulin, nor did we find them in the content of water or fat-soluble vitamins (Table 4). The glucogenicinsulin theory of milk fat depression assumes that tissues compete for nutrient use and that insulin causes nutrients to be diverted to adipose tissue at the expense of the mammary gland, as was observed in our experiments, however, without changing the level of insulin in the blood.

Studies of the past years confirm that the depression of fat in milk related to the change in the rumen biohydrogenic processes (the polyunsaturated fatty acids (PUFA) present in the fat of the diet are hydrogenated rumen bacteria), and not to changes in the rumen VFA. Under normal feeding conditions, very few unsaturated fatty acids reach the small intestine. The precursors of PUFA in the diets of dairy cows are the linoleic acid (C _18:2_) and linolenic acid (C_18:3_) contained mainly in plant lipids. A high concentration diet led to an increase of TFA in milk [26,38]. It has been demonstrated that conjugated linoleic acid (CLA) and trans-18:1 fatty acids (LC) resulting from the incomplete biohydrogenation of dietary polyunsaturated fatty acids (PUFA) in the rumen [3,39,40], or abomasal infusion [26,38,41], can significantly alter the synthesis of milk fat. Acetyl CoA carboxylase (ACA) has been identified as a limiting enzyme for the synthesis of fatty acids in the mammary glands [42]. The measured enzyme activity, together with tRNA for ASA, fatty acid synthetase, and sterol CoA desaturase in the mammary glands of cows, were markedly reduced in cows fed a diet that caused a depression of fat formation [9,39]. Unfortunately, no measurements were carried out in our experiments on CLA in rumen fluid, and, most importantly, CLA in rumen fluid was not altered by the diet intake. Therefore, it can only be assumed that the noted shifts in scar fermentation (a tendency to have lower pH (*p* = 0.09), a significant increase in propionate (*p* = 0.02), and a significant decrease in buffering capacity (*p* = 0.04)) could lead to a decrease in bio-hydrageneration capacity. However, this is only our guess. When milk fat is depressed, short-chain fatty acids (<C_16_) are primarily reduced. This indicates that trans fatty acids reduce the fat content in milk due to de novo synthesis. In our experiments, the fatty acid composition of milk fat did not differ in the two groups of cows. There was only a slight unreliable decrease in short and medium chain fatty acids in the milk of cows with low milk fat (54.4 ± 2.00 and 56.8 ± 1.48; *p* > 0.35) (Table 7). An increased content of fatty acids with an odd number of atoms (C_15_ and C_17_) was revealed in the milk of cows with a low fat content of milk (*p* < 0.05) (Table 7). An increase in the composition of milk fat with its low content of C_14_, C_16_, and C_17_ was also noted in other works [43].

In our experiment, cows of both groups received the same diet; however, the fat content of the milk was very different. Apparently, there are additional factors that allow some cows to withstand the feed load for ruminal microbiocenosis or a different response. The increased production of trans-10 and cis-12 CLA in the rumen does not provide a universal explanation for the decrease in milk fat during diet-induced milk fat loss, suggesting that other biohydrogenation intermediates may also be involved [24]. Further research is needed to characterize the structure and function of other biohydrogenation intermediates and to consider the contribution of broader changes to rumen lipid metabolism to provide a more universal explanation for diet-induced low-fat milk syndrome (milk fat depression).

## 5. Conclusions

In our experiment, cows of both groups received the same diet; however, the fat content of the milk was very different. A decrease in the release of energy with milk led to an increase in the deposition of body reserves, as a result of which the availability of milk synthesis increased, and the service period decreased. This was due to the metabolizable energy being directed to body reserves instead of milk fat. The study of the functioning of the scar and the concentration of the main metabolites and hormones in the blood did not reveal significant changes that would explain the decrease in fat excretion with milk. It was found that the low milk fat content was not associated with low acetate formation in the rumen and hormone levels. Further study of this issue is required, taking into account the formation of CLA.

## Figures and Tables

**Table 1:** Characteristics of cows in the experiment (50–52 days of lactation) (M ± SE; *n* = 10).

Item	Groups		Contrast (*p*-Value)
	Cows with Normal Milk Fat	Cows with Low Milk Fat	
live weight, kg	625 ± 23.7	629 ± 30.9	0.54
BCS	2.85 ± 0.2	2.95 ± 0.15	0.55
number of lactation	1.5 ± 0.2	1.6 ± 0.31	0.48
stage of lactation (days)	57.0 ± 6.53	58.0 ± 12.2	0.46
milk yield, kg/d	41.2 ± 1.79	39.5 ± 1.55	0.21
milk fat, %	3.94 ± 0.12	2.95 ± 0.14	0.0005
milk protein, %	3.18 ± 0.08	3.12 ± 0.07	0

**Table 2:** Composition of total mixed diet fed to cows.

**Composition**	**% of DM**
copped grass hay	1.6
haylage of perennial grasses	8.0
corn silage	23.9
compound feed	52.1
beer shot	7.8
soybean meal	3.3
molasses	2.9
sodium bicarbonate	0.1
tricalcium phosphate	0.15
salt plus trace mineralized salt and vitamins	1.9
**Chemical composition**	**(% of DM)**
OM	93.4
CP	16.5
NDF	33.1
forage NDF	19.7
ADF	17.5
total FA	3.86
starch	23.4
ME (MDJ/kg)	9.91

OM: organic matter, CP: crude protein, NDF: neutral detergent fiber, ADF: acid detergent fiber, FA: fatty acids, ME: metabolizable energy.

**Table 3:** Characteristics of the ruminal fluid of cows (M ± SE, *n* = 5).

Item	Groups		Contrast (*p*-Value)
	Cows with Normal Milk Fat	Cows with Low Milk Fat	
pH	6.95 ± 0.209	6.49 ± 0.207	0.09
Buffer capacity, mL/pH	12.5 ± 0.78	8.5 ± 1.20	0.043
NH_3_-N (mg/dL)	5.53 ± 2.73	4.48 ± 1.63	0.44
VFA			
Total (mmol/dL)	9.2 ± 1.37	10.7 ± 0.76	0.17
Individual (mol/100 mol)			
Acetate	65.6 ± 1.36	62.2 ± 1.74	0.1
Propionate	18.8 ± 0.57	25.2 ± 1.85	0.02
Butyrate	15.5 ± 1.50	12.4 ± 0.61	0.04
Bacterium 10^6^ mL^−1^	9.2 ± 0.50	9.7 ± 0.34	0.34
Protozoa, ×10^5^ mL^−1^	315 ± 35	251 ± 73.7	0.39
Amylolytic activity, E dL	34.2 ± 3.6	32.2 ± 0.74	0.47
Fibrolytic activity,%	12.8 ± 0.67	10.2 ± 0.65	0.07

**Table 4:** Biochemical parameters of the blood of the cows (M ± SE, *n* = 5).

Indicators	Groups		Contrast (*p*-Value)
	Cows with Normal Milk Fat	Cows with Low Milk Fat	
α-amine nitrogen, mmol/L	5.85 ± 0.48	6.03 ± 0.193	0.36
lipids, g/dL	0.48 ± 0.06	0.35 ± 0.07	0.28
glucose, mmol/L	3.57 ± 0.055	3.35 ± 0.207	0.31
VFA, mmol ь/L	1.99 ± 0.03	2.16 ± 0.02	0.01
β-hydroxybutyrate, mmoL/L	0.64 ± 0.15	0.45 ± 0.043	0.22
triacylglycerols, mcmol/L	118.4 ± 12.06	99.1 ± 12.4	0.24
NEFA, mmol/L	0.31 ± 0.015	0.23 ± 0.028	0.04
thiamine, mcg/mL	0.1 ± 0.006	0.088 ± 0.011	0.39
pyruvate, mcg/mL	8.6 ± 1.3	12.1 ± 1.36	0.117
pyruvate carboxylase, mmol/L/min	2.74 ± 0.17	3.35 ± 0.28	0.075
lactate dehyrogenase, mmol/L/min	21.1 ± 2.33	32.1 ± 1.21	0.001
acetoaldehyde, mcg/mL	3.1 ± 1.92	0.29 ± 0.11	0.277
α-tocopherol, mcg/mL	12.9 ± 3.23	8.6 ± 1.04	0.31
retinol, mcg/mL	0.33 ± 0.0035	0.29 ± 0.014	0.08
insulin, mked/mL	19.9 ± 0.56	20.03 ± 0.48	0.86
cortisol, nmol/L	382 ± 32.9	295 ± 37.2	0.128
thyroxine, ng/mL	3.34 ± 0.45	3.38 ± 0.18	0.94
triiodothyronine, nmol/L	271.3 ± 19.4	275.5 ± 11.3	0.86

**Table 5:** The efficiency of using ME in cows with different milk fat contents (M ± SE, *n* = 5).

Item	Cows with Normal Milk Fat	Cows with Low Milk Fat	Contrast (*p*-Value)
Metabolizable energy, MJ	265.1 ± 0.12	265.0 ± 0.11	0.49
Heat production, MJ	108.7 ± 1.94	102.5 ± 1.14	0.055
Heat production, % ME	41.0	38.7	
Milk energy, MJ	122.9 ± 5.12	106.7 ± 3.03	0.10
Energy retention, MJ	4.68 ± 8.26	30.8 ± 4.84	0.035
BCS	2.7 ± 0.15	2.85 ± 0.18	0.43

**Table 6:** Daily milk yield and milk composition in cows (days 80–82 of lactation) (M ± SE, *n* = 10).

Item	Groups		Contrast (*p*-Value)
	Cows with Normal Milk Fat	Cows with Low Milk Fat	
Milk yield, kg/d	39.0 ± 1.77	40.5 ± 1.25	0.21
Milk fat, %	4.1 ± 0.24	2.84 ± 0.13	0.00015
Milk protein, %	3.16 ± 0.05	3.16 ± 0.04	0.5
Lactose, %	5.08 ± 0.08	5.13 ± 0.06	0.48
Glucose, mM/L	0.34 ± 0.123	0.22 ± 0.065	0.41
Urea, mM/L	5.62 ± 0.337	5.12 ± 0.607	0.38

**Table 7:** Milk fat composition (g/100 g) (M ± SE, *n* = 5).

Variable	Cows with Normal Milk Fat	Cows with Low Milk Fat	Contrast (*p*-Value)
C _8:0_	1.09 ± 0.13	0.87 ± 0.09	0.23
C _10:0_	3.69 ± 0.39	3.17 ± 0.30	0.33
C _11:0_	0.32 ± 0.05	0.29 ± 0.04	0.72
C _12:0_	4.50 ± 0.47	3.98 ± 0.36	0.41
C _13:0_	0.08 ± 0.01	0.15 ± 0.03	0.07
C _14:0_	12.66 ± 1.10	12.07 ± 0.87	0.69
C _14:1_	0.51 ± 0.14	0.31 ± 0.03	0.26
C _15:0_	0.08 ± 0.03	0.27 ± 0.06	0.03
C _15:1_	0.15 ± 0.03	0.08 ± 0.02	0.07
C _16:0_	30.19 ± 0.91	29.92 ± 0.84	0.83
C _16:1_	1.63 ± 0.27	1.56 ± 0.15	0.81
C _17:0_	0.10 ± 0.01	0.18 ± 0.02	0.022
C _17:1_	0.17 ± 0.05	0.18 ± 0.02	0.82
C _18:0_	12.49 ± 0.76	11.99 ± 0.59	0.62
C _18:1_	27.66 ± 1.98	30.15 ± 1.98	0.40
C _18:2_	3.45 ± 0.39	3.43 ± 0.37	0.97
C _18:3_	0.92 ± 0.13	1.01 ± 0.09	0.59
C _20:0_	0.11 ± 0.02	0.13 ± 0.01	0.59
C _20:1_	0.08 ± 0.02	0.12 ± 0.01	0.17
C _20:4_	0.12 ± 0.03	0.14 ± 0.02	0.68
Total C_8–6_	56.8 ± 1.48	54.4 ± 2.00	0.35
Total C_16:1–20_	43.2 ± 1.48	45.6 ± 2.00	0.17

**Table 8:** Dynamics of milk yield by months.

Groups	Milk Yield, kg/d^-^							MILK Fat, %						
	Days of Lactation						Average for 5 Months of Lactation	Days of Lactation						Average for 5 Months of Lactation
	50	80	110	140	170	200		50	80	110	140	170	200	
1	41.2 ± 1.79	39.0 ± 1.77	37.2 ± 2.71	35.3 ± 1.45	33.0 ± 2.08	30.0 ± 2.51	35.95 ±	3.94 ± 0.12	4.1 ± 0.24	3.52 ± 0.06	3.4 ± 0.17	3.72 ± 0.23	3.72 ± 0.18	3.7 ± 0.09
2	39.5 ± 1.55	40.5 ± 1.25	39.6 ± 1.72	33.8 ± 0.8	30.6 ± 2.31	32.4 ± 3.51	36.1 ±	2.95 ± 0.14	2.84 ± 0.13	2.91 ± 0.09	3.23 ± 0.21	4.0 ± 0.28	3.65 ± 0.26	3.3 ± 0.11
p	0.21	0.21	0.24	0.21	0.24	0.29	0.47	0.0005	0.001	0.0006	0.28	0.24	0.41	0.01

## Data Availability

The data that support the findings of this study are available from the corresponding author upon reasonable request.
